# The Infant Gut Microbiota and Risk of Asthma: The Effect of Maternal Nutrition during Pregnancy and Lactation

**DOI:** 10.3390/microorganisms8081119

**Published:** 2020-07-25

**Authors:** Naser A. Alsharairi

**Affiliations:** Heart, Mind & Body Research Group, Menzies Health Institute Queensland, Griffith University, Gold Coast 4215, Australia; naser.alsharairi@gmail.com

**Keywords:** maternal diet, infant, gut microbiota, asthma, pregnancy, lactation

## Abstract

Research has amply demonstrated that early life dysbiosis of the gut microbiota influences the propensity to develop asthma. The influence of maternal nutrition on infant gut microbiota is therefore of growing interest. However, a handful of prospective studies have examined the role of maternal dietary patterns during pregnancy in influencing the infant gut microbiota but did not assess whether this resulted in an increased risk of asthma later in life. The mechanisms involved in the process are also, thus far, poorly documented. There have also been few studies examining the effect of maternal dietary nutrient intake during lactation on the milk microbiota, the effect on the infant gut microbiota and, furthermore, the consequences for asthma development remain largely unknown. Therefore, the specific aim of this mini review is summarizing the current knowledge regarding the effect of maternal nutrition during pregnancy and lactation on the infant gut microbiota composition, and whether it has implications for asthma development.

## 1. Introduction

Asthma is considered one of the most widespread chronic respiratory diseases of childhood, with hospitalization rates reportedly having increased for children under the age of 5 years over the last two decades [1]. The body of evidence around asthma development in young children suggests that genetic predisposition [2,3,4], environmental exposures [2,5] as well as the gut microbiome [6,7,8,9] have a strong influence.

The maternal gut microbiota promotes the microbiota colonization of the infant gut prior to birth in utero, after delivery and then through breastfeeding [10,11,12]. Gut microbial colonization during early life represents a critical window of offspring immune system development [13]. According to the sterile womb paradigm, the gut microbiota are transferred from mother to infant prior to or during delivery and breastfeeding via a vertical pathway, and from the environment after delivery via a horizontal pathway [14,15]. Early-life microbial dysbiosis of the gut has been linked to an increased risk of asthma in the first years of life [16,17,18]. Such a link is significantly modified by the mode of birth delivery, antibiotic exposure and maternal body mass index (BMI), asthma and stress [6,16,18,19,20,21,22,23]. However, pre- and postnatal pet exposure may influence the infant gut microbiota and the development of their immune systems, with a subsequent reduced risk of asthma [24].

The maternal diet has been found to be a significant factor that influences the gut microbiota and potentially the development of asthma in offspring [25]. There is apparently an additional value obtained from animal models in understanding the mechanisms by which maternal dietary patterns during pregnancy influence asthma development in offspring via the maternal gut microbiota and their metabolites [21,26,27]. However, our understanding of the aforementioned mechanisms is incomplete and further long-term studies in humans are needed. In addition, the mechanisms by which maternal dietary patterns and breast milk microbiota alter the offspring gut microbiota, which may in turn underlie asthma, remain underdeveloped. Indeed, the protective role of breastfeeding against asthma in childhood is still a controversial matter. This controversy arises because there are inconsistent results from previous studies [19,28]. Recently, the modulation of gut microbiota composition by probiotics and prebiotics during pregnancy and lactation has been proposed as a potential dietary strategy to reduce the risk of asthma in offspring. Despite evidence of alterations in maternal gut bacteria, a systematic review and meta-analysis of a randomized controlled trial (RCT) reported no benefit of probiotic supplements during pregnancy and early life for asthma or wheeze prevention in children [29]. Because of the low certainty of the evidence, the World Allergy Organization panel suggests not recommending prebiotic supplementation in both pregnant and lactating women for the prevention of asthma and allergic diseases in children [30].

The maternal gut microbiota progressively changes throughout pregnancy and significantly determines what is transmitted to the offspring and how the offspring gut microbiota eventually develops [31]. Diet is considered a major driver of the maternal gut microbiota changes [31] and therefore, in human studies, maternal dietary intake in gestation is associated with alterations in the offspring gut microbiota as well [32,33,34]. In particular, the type of dietary fat in the diet of pregnant mothers can negatively affect the trajectory of the offspring microbiota. It has been found that a high-fat maternal diet during gestation is associated with lower levels of the genera Bacteroides in the offspring gut [32].

Breastfeeding has a significant effect on the alterations in the gut microbiota following birth either by infant exposure to milk microbiota or by breast milk factors interacting with the maternal and infant gut microbiota such as human milk oligosaccharides (HMOs) [35]. The composition and diversity of breast milk microbiota are influenced by several maternal factors such as mode of delivery, gestational age, lactation stage, maternal BMI, mode of feeding and antibiotic exposure [36,37,38,39,40,41,42,43,44,45]. A few compelling studies have been undertaken which do indeed demonstrate that maternal dietary nutrient intake strongly influences breast milk microbiota. Further studies are needed to understand how maternal nutrition during pregnancy and lactation alter the infant gut microbiota, and whether this interaction increases the risk of asthma. Therefore, this mini review aims to summarize the existing evidence demonstrating the effect of maternal nutrition during pregnancy and lactation on the infant gut microbiota composition, and whether it influences asthma risk.

## 2. Gut Microbiota in Early Life and Its Relationship with Asthma Development

The first large wave of microbes to colonize the gut occurs following birth and the gut continues to acquire microbes during the transition between infancy and early childhood [12]. The early gut microbial colonization plays a significant role in the maturation of the metabolic, endocrine and immune systems [46]. The gut microbiota during the first three years of life has taxonomic and functional compositions different to that in adults, and the gut of some infant may exhibit lower microbial diversity than in others [47]. Following birth, the gut microbiota is predominantly colonized by Enterobacteriaceae and Staphylococcus genera [11]. During breastfeeding, the gut microbiota are dominated by Bifidobacterium genera which have the ability to metabolize HMOs [11,48]. HMOs are soluble complex carbohydrates and are thought to play a crucial role in increasing the abundance of Bifidobacteria which protect against pathogenic infection and alter bacteria-host interactions [42,49,50]. The time of weaning, the initiation of solid foods results in the establishment of microorganisms, represented by the genera Bacteroidetes and Firmicutes [11,51], species known to disassemble complex plant-derived polysaccharides [52]. The introduction of food items with protein and fiber contents (e.g., rye bread, meat, cheese, animal fat) increased the relative abundance of *Ruminococcaceae* and *Lachnospiraceae* spp. in the infant gut [53]. By around three years of age, the gut microbiota are colonized by microbial-enriched genes, most belong to the genera Prevotella, Veillonella, Ruminococcus, Clostridium, Bacteroides and Firmicutes, species involved in carbohydrate metabolism, xenobiotic degradation and vitamin B synthesis [11,47].

There is strong evidence that early-life gut microbial exposure plays a significant role in the development of childhood asthma [17]. The link between the early life dysbiosis of the gut microbiota and the subsequent development of asthma has been well-established in large-cohort studies [16,17,18]. It has also become clear through findings from epidemiological studies and reviews that this link is conceivably influenced by a wide range of perinatal factors. These findings suggest that maternal asthma during pregnancy [18,23], pre-gestational BMI [23], delivery mode (vaginal, cesarean) [6,16,19,20], breastfeeding mothers with a history of atopic conditions [19], maternal stress [19,22] and antibiotic exposure [6,19,21] are considered the main modifiers of the infant gut microbiota contributing to the development of asthma. 

Maternal diet has also been considered a key factor that influences asthma development in offspring through the maternal gut microbiota and its metabolites’ modulating effects [25]. There are substantial animal mechanistic studies, which demonstrate that diet and microbial exposure during pregnancy influence asthma development in offspring [26]. However, our understanding at this point is still incomplete in human studies. Evidence in animal models demonstrates that the gut microbiome transferred through the placenta produces diverse metabolites, which act as significant mediators of fetal immune development [21]. Short-chain fatty acids (SCFAs), propionate, acetate and butyrate, are the main metabolites generated by the microbial fermentation of complex plant carbohydrates derived from microbiota-accessible carbohydrates (MACs), which represent the major energy sources for gut bacteria [21,27]. SCFAs influence T lymphocytes and dendritic cells through their binding to protein-coupled receptors and the direct inhibition of histone deacetylase, thereby promoting the differentiation of helperT cells (Th1, Th2). The Th2 asthmatic phenotype plays a pivotal role in increasing eosinophils, Immunoglobulin E (IgE) production, and the production of inflammatory cytokines. These cytokines enhance the allergic immune system and increase the risk of asthma in offspring [21,26]. Changes in dietary patterns during pregnancy may affect the microbial composition and diversity in the gut as well as the production of SCFAs [21]. A low-MAC diet is found to decrease bacterial diversity and SCFA production, which may hinder the functioning of regulatory T cells and lead to inhibited immunoglobulin A and G (IgA, IgG) production [27]. Data from human research has shown that a high maternal fiber intake during late pregnancy increases acetate (but not propionate or butyrate) levels in serum, which leads to a reduced risk of coughing and wheezing symptoms in the offspring’s first year of life [54].

## 3. Breastfeeding as a Key Pillar of Asthma Prevention by Microbiota Shaping

Breastfeeding has a wide range of positive short-and long-term health benefits for mothers and infants, including a protective role against neurological disorders, cancers and obesity in infants and diabetes/cardiovascular risk in mothers [55]. Despite its known protective role, not all studies have reported a protection against childhood asthma [19,28]. Breast milk in its complex composition contains several bioactive components such as cytokines, immunoglobulins, chemokines, polyunsaturated fatty acids, eosinophil-derived granular proteins, antigens and polyamines that stimulate infant immune system development [28]. Since gut microbial dysbiosis in early life is found to be associated with asthma development [17], breast milk would be an optimal nutritional solution for modulating the infant gut microbiota, which may therefore reduce the long-term asthma risk. Breast milk has the potential to shape the gut microbiota after birth by exposure of the infant to the milk microbiota or by breast milk-modulating factors such as HMOs, which can affect microbiota growth and metabolism [35]. Breast milk is a source of probiotics (milk microbiota) and prebiotics (HMOs) which play a role in increasing the colonization of beneficial bacteria in the infant gut [50]. An analysis of breast milk using culture-dependent and independent techniques has revealed that lactic acid bacteria, *streptococci* and *staphylococci* spp. are the most abundant beneficial bacteria to the offspring gut [56]. Indeed, it remains unclear how the milk microbiota are established. An entero-mammary pathway has been proposed to explain the origin of bacteria in milk. This pathway involves bacterial translocation through the intestinal lumen by internalization in the maternal dendritic cells or macrophages, which is then taken up into the lactating mammary gland through blood/lymphatic circulation [56].

## 4. Search Methodology

The PUBMED/MEDLINE database was searched up to June 2020 to identify pertinent studies. Studies of potential interest published in English were selected for inclusion if they addressed the effect of maternal nutrition on the infant gut/milk microbiota composition, and/or their consequences on asthma development. Relevant articles were identified using the combination of the following search terms: (nutrition OR diet OR dietary pattern OR dietary quality) AND (gut microbiota OR milk microbiota) AND (pregnancy OR lactation). The search was limited to human studies. A total of 712 articles were assessed using the specified searches. As a result, six studies met the search criteria. The selected studies are provided in the following section and a summary is presented in Table 1.

## 5. Effect of Maternal Nutrition during Pregnancy and Lactation on Infant Gut and Milk Microbiota Composition

### 5.1. Maternal Nutrition during Pregnancy 

The pregnancy period is marked by substantial immunological, metabolic and hormonal changes which appear to be significant factors in modulating the mother’s gut microbiota composition and function [59]. The effect of maternal nutrition during pregnancy on infant gut microbiota composition alterations has been widely reported in animal models; however, evidence from cohort studies in humans is limited [59,60,61]. One study [32] reported associations between a maternal high-fat diet intake and the abundance of genera Bacteroides and Enterococcus in the infant gut. Another study [33] showed that the associations between maternal dietary patterns and the infant gut microbiota at 6 weeks postpartum differ according to the mode of birth delivery. In a recent study [34], consuming the recommended amount of fish was found to be associated with Bifidobacterium dominance in the infant gut.

### 5.2. Maternal Nutrition during Lactation 

Maternal nutrition may influence the milk microbiota composition and diversity directly [40,57] or indirectly [43] through alterations in the maternal gut microbiota diversity. Few studies on the effects of maternal nutrition during lactation on the milk microbiota composition have been conducted. Research evidence has revealed that variations in maternal macro- and micronutrients intake impacted on the relative abundance of several bacterial taxa in milk 6 months postpartum [40]. A recent study [57] has demonstrated that maternal dietary nutrient intake influences the composition of milk microbiota, and such effect appears to be different between pregnancy and lactation. Another study [58] found that a maternal high-fat diet throughout breastfeeding altered both the milk microbiota and HMO. Collectively, these studies indicate that maternal nutrition may alter the microbial composition of breast milk. The mechanisms whereby maternal nutrition influences the milk microbiota are not yet fully understood. It is likely that both probiotic (bacteria in the diet) and nutrient intake influence the microbial composition present in the maternal gastrointestinal (GI) tract and these may enter the mammary glands through the entero-mammary route [62]. 

### 5.3. Effect Modification 

Among the key factors worthy of explanation for associations between maternal nutrition and alterations in infant gut and breast milk microbiota is obesity. Previous studies have shown evidence indicating that obesity-associated maternal gut microbiota may alter the infant gut microbiota towards a state of dysbiosis and induce later-life obesity. In support of this fact, lower levels of *Bacteroides* spp. and *Bifidobacterium* spp. have been found to be associated with weight gain in obese infants compared with their normal weight counterparts [63,64,65,66]. In addition, a significantly less diverse microbiota in breast milk has been demonstrated in obese breastfeeding mothers, corroborating the conclusion that obesity influences the milk microbiota [36,40,43,62]. The abundance of the Bacteroides genera decreased significantly in milk produced by obese mothers [40], who consumed high-fat diets during pregnancy and lactation [32]. Obesity alters the maternal microbiota composition during pregnancy and lactation, and this may have a significant impact on asthma risk in the first years of life [63,67]. The immune system reacts to changes in microbiota composition, and gut microbiota depleted of *Proteobacteria* spp. in infants of mothers with obesity could result in alterations in immune profiles, leading to an increased risk of developing inflammatory diseases [67]. Infants born to obese mothers or fed a high-fat diet showed a disturbed gut microbiota that is not entirely corrected by offering a specific diet later by complementary feeding. This has profound clinical implications for maternal diet-induced alterations to the maternal gut microbiota which, in turn, could have a lasting impact on the infant gut microbiota composition, thus making children more susceptible to inflammatory diseases [65]. Further long-term human dietary interventions are needed to determine if specific changes in maternal dietary patterns during both pregnancy and lactation induce permanent alterations in the infant gut microbiota, and to delineate if this results in reduced risk of asthma later in life. A direct effect of maternal nutrition on the milk microbiota with subsequent development of asthma, and as a result of the influence of obesity, has not yet been uncovered. Given that milk microbiota are associated with obesity [36,40,43,62], a skewed microbial milk composition could be a potential factor contributing to shifts in the microbial composition of the infant gut, and might contribute to asthma. Therefore, understanding the effect of milk microbiota on obesity-related asthma could facilitate the development of new avenues for dietary interventions during both pregnancy and lactation to reduce the risk of asthma.

Maternal early life factors (mode of delivery, antibiotics use and mode of feeding) may also modify such association. Obesity interacting with maternal and early life factors may alter the gut/milk microbiota and increase the susceptibility of children to develop asthma later in life. Infants born by cesarean (C-section) are at an increased risk of food allergies [68], obesity [69] and asthma [70]. The breast milk of mothers who had delivered by elective C-section was associated with reduced relative abundances of beneficial microbes [36,37,38,39,44]. The use of antibiotics for C-section delivery increases the susceptibility to gut dysbiosis and obesity later in life [69]. Both prenatal and postnatal exposure to antibiotics increases the risk of asthma in childhood [6,19,21]. Mothers following elective C-section delivery without intrapartum antibiotic use exhibited significantly lower milk microbiota richness and diversity compared to mothers who used antibiotics and delivered vaginally [44].

Formula feeding is associated with a higher risk of obesity and asthma than breastfeeding [71,72]. Compared to breastfed infants, formula-fed infants are exposed to a mix of nutrients and a more complex form of carbohydrates, causing colonization with diverse gut microbes [14]. Infants who were fed exclusively formula/breastmilk supplemented with formula and born by C-section had a less diverse gut microbial community [73,74]. Infants who were not breastfed but who were exposed to antibiotics and born by C-section delivery displayed significantly decreased relative abundances of gut microbial taxa [75]. Exclusive breastfeeding over mixed feeding can potentially restore the gut microbiota dysbiosis of C-section born infants [76].

## 6. Conclusions

The maternal gut microbiota promotes the microbiota colonization of the infant gut throughout pregnancy and later in life. Therefore, it is likely that maternal gut microbiota during both pregnancy and lactation influences the infant gut microbiota via maternal nutrition. The exact mechanisms are yet to be fully understood, and further long-term follow-up studies of pregnancy and lactation cohorts are needed. While few prospective studies have found associations between maternal nutrition during pregnancy and alterations in the infant gut microbiota, no studies have found that such associations were related to an increased risk of asthma later in life. There is also a paucity of human data examining the associations between maternal dietary nutrient intake during lactation and alterations in milk microbiota composition. However, the effect of such associations on the infant gut microbiota and, furthermore, their consequences in relation to asthma, still remain unclear and require more comprehensive investigations.

This review suggests that maternal life factors (e.g., mode of delivery, antibiotics use and mode of feeding) may modify the associations between maternal nutrition and the infant gut/milk microbiota composition. Overall, maternal dietary patterns during both pregnancy and lactation influence maternal obesity, which directly influences infant gut/milk microbiota. Maternal nutrition may indirectly influence the infant gut/milk microbiota through maternal early life factors. These factors may affect maternal obesity and could directly alter the milk microbiota by influencing the infant gut microbiota. Further studies are needed to examine the potential modification effect caused by these factors in the association of maternal nutrition–infant gut microbiota with subsequent asthma development.

## Figures and Tables

**Table 1:** Alterations of infant gut and/or milk microbiota associated with maternal nutrition.

Design and Subject Characteristics.	Maternal Nutrition	To Indicate if Microbiota Changes are Seen in Infants’ Gut or Milk Microbiota	Ref.
Prospective cohort study 81 pregnant women 82 women at delivery and 6-wk postpartum	High-fat diet	Third trimester of pregnancy = Enterococcus↑, Bacteroides↓ At delivery and beyond = Bacteroides↓	[32] ^a^
Prospective cohort study 97 vaginal delivery 48 cesarean section delivery	Mediterranean (MED) Dairy Fish and seafood Fruit Red and processed meat	Vaginal delivery MED = Streptococcus↑, Clostridium↑, Bacteroides↓, *Escherichiacoli*↓, Ruminococcus↓ Dairy = Clostridium↑, Staphylococcus↑, *Lachnospiraceae*↓ Fish and seafood = Streptococcus↑, Bacteroides↓ Fruit = Clostridium↑, Bifidobacterium↓ Cesarean delivery MED = Enterococcus↑, *Lachnospiraceae*↑, *Escherichiacoli*↓, Streptococcus↓, Blautia↓ Dairy = *Enterobacteriaceae*↑, *Escherichiacoli*↑, Bifidobacterium↓, Pseudomonas↓, Bacteroides↓, Corynebacterium↓ Fish and seafood = Bacteroides↑, Clostridium↓, Streptococcus↓ Red and processed meat = Bifidobacterium↑, *Escherichiacoli*↑, Enterococcus↑	[33] ^a^
Prospective cohort study 114 infants	Fish	Third trimester of pregnancy = Bifidobacterium↑	[34] ^a^
Prospective cohort study 21 lactating women	Macro- and micronutrients	Energy and protein = Gemella↑ SFA and MUFA = Corynebacterium↓ Total carbohydrates, total disaccharides and lactose = Firmicutes↓ Insoluble fiber = Rothia↑ Pantothenic acid = Streptococcus↓ Riboflavin and calcium = Veillonella↑ Thiamine, niacin, folate, vitamin B-6 and chromium = Lactobacillus↓	[40] ^b^
Cross-sectional study 94 lactating women	Nutrients	Pregnancy: vitamin C = Staphylococcus↑ Lactation (1-month post-partum): PUFA & linoleic acid = Bifidobacterium↑, sugars = Pseudomonas↓, vitamin B9 = Pseudomonas↑, Thiamine & vitamin B9 = Enterococcus↓	[57] ^b^
Prospective cohort study 14 lactating women	High-fat diet	*Erysipelotrichaceae*↑, Roseburia↑	[58] ^b^

^a^ Studies suggest that maternal nutrition lead to a change in the infant gut microbiota. ^b^ Studies suggest that maternal nutrition lead to a change in the milk microbiota. ↑ reflects positive associations, ↓ reflects negative association.
